# High Lipid Yield and Efficient Biodiesel Production from *Fusarium solani* 102F Using Waste Eggshell as Biocatalyst

**DOI:** 10.3390/microorganisms14102272

**Published:** 2026-10-06

**Authors:** Doaa M. A. Khalil, Mostafa Abdelrahman, Ha Duc Chu, Sudisha Jogaiah, Lam-Son Phan Tran, Noha M. Kamel

**Affiliations:** 1 Botany Department, Faculty of Science, Aswan University, Aswan 81528, Egypt; doaa.khalil30@sci.aswu.edu.eg; 2 Institute for One Health and Innovation, Texas Tech University, Lubbock, TX 79409, USA; mosabdel@ttu.edu; 3 Faculty of Agricultural Technology, University of Engineering and Technology, Vietnam National University Hanoi, Hanoi City 100000, Vietnam; cd.ha@vnu.edu.vn; 4 VinUni Bigdata Research Institute, VinUniversity, Hanoi City 100000, Vietnam; 5 Department of Environmental Science, Central University of Kerala, Tejaswini Hills, Periye, Kasaragod 671 325, Kerala, India; jsudish@cukerala.ac.in; 6 Institute of Genomics for Crop Abiotic Stress Tolerance, Department of Plant and Soil Science, Texas Tech University, Lubbock, TX 79409, USA; son.tran@ttu.edu

**Keywords:** microbial biodiesel, eggshell transesterification, *Fusarium solani*, gas chromatography mass spectrometry

## Abstract

Oleaginous fungi offer a sustainable alternative to plant-based feedstocks for biodiesel production, yet their industrial potential remains limited due to high processing costs and strain-dependent performance. Here, we report that the endophytic fungus *Fusarium solani* 102F, isolated from a drought-tolerant legume tree in a hot–arid region, accumulates lipids and serves as a promising microbial platform for biodiesel production. Through systematic screening of nine endophytic isolates, *F. solani* 102F emerged as the top lipid producer with low biomass accumulation. Stepwise preliminary screening of culture conditions, including incubation time, agitation, pH, temperature, carbon and nitrogen sources, and salt, significantly increased biomass and lipid production. The highest FAME was achieved with glucose and casein as the best carbon and nitrogen sources, respectively, at 25 °C, pH 5.0, and 200 rpm shaking over seven days. Moderate salt level further boosted lipid accumulation compared with high salt level. Direct transesterification of *F. solani* 102F biomass yielded 15.8% FAMEs, supporting its potential as a microbial feedstock for biodiesel production. Importantly, we demonstrate that waste eggshell-derived CaO acts as a low-cost, effective heterogeneous biocatalyst for the transesterification of extracted fungal lipids from *F. solani* 102F, increasing biodiesel yield from 15.8% in the non-eggshell treatment to 27.02%, corresponding to a 71% relative increase. The fatty acid methyl esters resulting from transesterification with ES-derived CaO were analyzed by GC–MS, revealing a transesterified lipid profile enriched in C16–C18 monounsaturated chains, making them highly suitable for biodiesel production. This integrated approach, combining a robust fungal strain with waste eggshell-derived catalyst, provides a potential route for microbial biodiesel production, advancing circular bioeconomy strategies for renewable energy.

## 1. Introduction

The sustainability of first-generation feedstock-derived biodiesel is increasingly questioned because of its heavy use of land and water resources. This has driven more interest toward microbial alternatives for biodiesel production [1]. Biodiesel has become a promising alternative to petroleum-based fuels, mainly due to its biodegradability and lower greenhouse gas emissions [2,3]. Unlike plants, oleaginous fungi grow quickly, are independent of seasonal and climatic variations, and can turn low-cost industrial residues into triacylglycerols (TAGs) [4,5]. Oleaginous fungi can accumulate substantial amounts of lipids up to 20–50% (*w*/*w*) of their total dry biomass, especially under nitrogen-limited conditions [6,7]. *Mortierella alpina* remains a leading fungus for high-level lipid production, often exceeding 50% lipid content under nitrogen limitation [8]. *Rhizopus oryzae* B97 can reach up to 35% lipids when grown on cheap substrates, such as industrial sludge or lignocellulosic waste [2,9]. The fatty acid profiles of these fungi are rich in palmitic, stearic, and oleic acids, which closely match those of premium oils and are fully compatible with biodiesel standards [10,11].

Lipid accumulation in oleaginous fungi is primarily regulated by cultivation conditions, such as carbon-to-nitrogen ratio, pH, and aeration [12]. Under nitrogen limitation and high C/N ratios, the fungi shift their metabolism toward TAG production, which is necessary for lipid-rich biomass [13]. The transesterification of the fungal TAGs with short-chain alcohols results in the production of fatty acid methyl esters (FAMEs) that are fully compatible with conventional diesel engines [14]. Chemical catalysts remain the most popular option for this step, but researchers have been increasingly seeking reusable and low-waste alternatives. Metal–organic framework (MOF) catalysts, such as Na-Ni-MOF and Cu-benzenetricarboxylate, can achieve FAME yields in fungi up to 99% and are free from leaching and separation problems associated with homogeneous catalysts [15,16]. Waste-derived catalysts, such as CaO from chicken eggshell (ES), also improved transesterification and produced biodiesel yields in fungi up to 99.07% under the optimized methanol-to-oil ratio and catalyst loading [17,18]. Thus, the use of lipid-rich oleaginous fungi, low-cost waste substrates, and heterogeneous catalytic systems together make biodiesel production economically viable and reduce dependence on fossil fuels [11]. Despite recent advances, fungal biodiesel production still faces major challenges. FAME vary widely among strains, strain performance depends heavily on growth conditions, and there is a natural trade-off between biomass growth and lipid accumulation. Together, these issues continue to limit industrial scalability. Therefore, finding high-lipid fungal strains, fine-tuning growth conditions, and applying lipid-enhancing cheap biocatalysts are key parameters for making fungal biodiesel production competitive.

In this study, we hypothesize that lipid accumulation varies among oleaginous fungi and that suitable medium and growth conditions can increase lipid production in fungi. To test this hypothesis, we pursued the following objectives: (i) screen nine diverse fungal species isolated from a drought-tolerant legume tree grown in arid regions for biomass and lipid productivity, (ii) select the best fungal species associated with high lipid production and perform comparative expression analysis of the fatty acid synthase 1 (FAS1) gene between high- and low-lipid species to provide preliminary insights into lipid biosynthesis, and (iii) optimize cultivation conditions for the selected high-performing *Fusarium solani* 102F strain by evaluating temperature, agitation, pH, incubation time, and different C and N sources with potential metabolic modulators, such as salinity stress. We then evaluated the chicken ES-derived CaO catalyst for the transesterification of lipids into biodiesel in the 102F strain, with the aim of assessing its effectiveness in converting fungal lipids into FAMEs. To evaluate the lipid profile and demonstrate its suitability for biodiesel production, gas chromatography–mass spectrometry (GC-MS)-based lipid profiling was performed. Overall, this study identified a *Fusarium solani* strain that produces the highest FAME and provided its systematic characterization to demonstrate its potential use for biodiesel production.

## 2. Materials and Methods

### 2.1. Isolation, Maintenance, and Characterization of Endophytic Fungi

Endophytic fungi were isolated from the leaves of healthy *Dalbergia sissoo* specimens at Aswan University, Egypt. Tissue segments (~5 × 5 mm) were surface-sterilized sequentially with 70% ethanol (Sigma-Aldrich, St. Louis, MO, USA) for 1 min and 2.5% sodium hypochlorite (Sigma-Aldrich, St. Louis, MO, USA) for 2 min, followed by rinsing three times in sterile distilled water. Following surface sterilization, segments were then plated on potato dextrose agar (PDA) supplemented (PDA; HiMedia Laboratories, Mumbai, India) with 50 µg mL^−1^ chloramphenicol (Sigma-Aldrich, St. Louis, MO, USA) and incubated at 28 °C in the dark for 7–14 days until fungal hyphae emerged from the tissue edges. Emerging hyphal tips were sub-cultured onto fresh PDA to obtain pure isolates. Pure fungal isolates were maintained on PDA slants at 4 °C for short-term use and as spore suspensions in 25% (*v*/*v*) glycerol (Sigma-Aldrich, St. Louis, MO, USA) at −20 °C for long-term preservation. Prior to experiments, isolates were revived by sub-culturing on PDA at 28 °C for 7 days to ensure vigorous growth and standardized inoculum. Isolates were initially characterized morphologically by macroscopic colony traits (color, texture, pigmentation, and radial growth rate) and microscopic features (hyphal structure, conidiophore/conidia morphology, and fruiting bodies) on PDA after 7 days at 28 °C, using keys from Moubasher [19], Gilman [20], and Kim et al. [21].

### 2.2. Screening for Endophytic Lipid Production

To identify fungal isolates for sustainable biodiesel production, nine isolated endophytic fungi (*Aspergillus flavus*, *Aspergillus oryzae*, *Aspergillus terreus*, *Emericella nidulans*, *Fusarium dimerum*, *Fusarium oxysporum*, *Fusarium solani*, *Penicillium oxalicum*, and *Penicillium purpurogenum*) were screened for biomass yield and lipid accumulation potential in Czapek-Dox broth medium. The modified Czapek-Dox broth composition consisted of 30.0 g L^−1^ glucose (Sigma-Aldrich, St. Louis, MO, USA), 1.0 g L^−1^ NaNO_3_ (Sigma-Aldrich, St. Louis, MO, USA), 1.0 g L^−1^ KH_2_PO (Sigma-Aldrich, St. Louis, MO, USA), 0.5 g L^−1^ MgSO_4_·7H_2_O (Sigma-Aldrich, St. Louis, MO, USA), and 0.01 g L^−1^ FeSO_4_·6H_2_O (Sigma-Aldrich, St. Louis, MO, USA), with the initial pH adjusted to 6.5. A 5 mm agar disc from a 5-day-old PDA culture was inoculated into 100 mL sterile Czapek-Dox broth and incubated at 28 °C with 200 rpm shaking for 7 days. Fungal biomass was harvested by filtration (Whatman No. 1 filter paper, 15 cm diameter), washed with deionized water, and dried in a hot air oven (47900 Furnace, Nabertherm, Germany) at 50 °C for 24 h to constant weight for dry biomass determination [22,23]. Total lipids were extracted from the fungal isolates by a modified Folch method [24]. In brief, 100 mg of dried fungal biomass was vortexed for 5 min in 10 mL of chloroform: methanol (2:1, *v*/*v*) (Merck, Darmstadt, Germany). The mixture was centrifuged at 3000× *g* for 15 min at room temperature. The lower chloroform-rich lipid phase was carefully transferred into a pre-weighed glass vial. Residual solvent was evaporated under reduced pressure at 40 °C using a rotary evaporator (HAHN SHIN-3001, Bucheon, Gyeonggi-do, South Korea) according to Sulaiman et al. [25] and Youssef et al. [23]. The vial was re-weighed to determine gravimetric lipid content. FAME percentage was calculated as the percentage of extracted lipids relative to the initial dry biomass weight using the following equation:

Yield of lipid (%) = [Lipid content (g L^−1^)/weight of dried biomass (g L^−1^)] × 100

To confirm gravimetric results, total lipid concentration was quantified using a modified sulfo-phospho-vanillin colorimetric assay [26]. Absorbance was measured at 530 nm using a T60U UV-Vis spectrophotometer (PG Instruments Ltd., Beijing, China) and lipid content was interpolated from a triolein standard curve and expressed as % dry biomass.

### 2.3. Molecular Identification of Selected Fungal Isolates

Fungal isolates exhibiting high biodiesel production were selected for molecular identification using ribosomal DNA sequencing. Mycelia were cultured on potato sucrose agar at 25 °C for 7 days. Biomass was suspended in 100 μL sterile distilled water and boiled at 100 °C for 15 min prior to inactivating the fungal isolate. The internal transcribed spacer (ITS) region was amplified using universal primers ITS1 (5′-TCCGTAGGTGAACCTGCG G-3′) and ITS4 (5′-TCCTCCGCTTATTGA TATGC-3′), and sequencing was performed at SolGent (Daejeon, South Korea). Resulting sequences were queried against the National Center for Biotechnology Information (http://www.ncbi.nlm.nih.gov) using BLAST (version 2.17.0+). Phylogenetic relationships were inferred by aligning query sequences with reference sequences from GenBank using ClustalW implemented in MegAlign software version 5.05 (DNASTAR Inc., Madison, WI, USA). The obtained sequence was deposited in NCBI GenBank under accession number PZ374814.

### 2.4. FAS1 Gene Expression Analysis in Selected Fungal Isolates

Total RNA was extracted from fungal biomass of the superior *F. solani* 102F and inferior *A. oryzae* lipid-producing strains using TRIzol reagent (Thermo Fisher Scientific, Waltham, MA, USA), followed by DNase I treatment (DNase I, RNase-free; Thermo Fisher Scientific, Cat. EN0521). RNA quantity and purity were assessed using a Quawell Q9000 microvolume spectrophotometer (Quawell, Rocklin, CA, USA). First-strand cDNA was synthesized from 1 μg total RNA using the RevertAid First Strand cDNA Synthesis Kit (Thermo Fisher Scientific, Cat. K1622) with oligo(dT) primers, following the manufacturer’s protocol. Quantitative real-time PCR was performed on a qTOWER^3^ G Real-Time PCR Thermal Cycler (Analytik Jena GmbH, Jena, Germany) (using ViPrime PLUS Taq qPCR Green Master Mix I (Vivantis Technologies, Shah Alam, Selangor, Malaysia; Cat. QLMM12)). The *FAS1* gene was amplified using forward 5′-GTTCCACGGCACATCGACTA-3′ and reverse 5′-CAACCGTTAAGCATCCAGGC-3′ primers, while *β-tubulin* forward 5′-TGGGCCTCTTTGACATCTTC-3′ and reverse 5′-GCCGTAACCGGATCTTGTAG-3′ primers were used as a reference gene based on its commonly reported stability in fungal systems. Relative expression levels were calculated using the comparative threshold cycle (^ΔΔ^Ct) method from three biological replicates.

### 2.5. Preliminary Screening of Lipid Production by Improvement of Culture Nutritional and Environmental Parameters Under a Sequential OFAT Screening Strategy

To improve biodiesel production from the superior lipid-producing *F. solani* 102F isolate, nutritional and environmental parameters affecting fungal biomass and lipid accumulation were evaluated sequentially using a single-factor-at-a-time (OFAT) screening approach. All experiments were performed with independent biological replicates using 100 mL Czapek-Dox broth (mentioned in Section 2.2) in 250 mL Erlenmeyer flasks and inoculated with a 5 mm fungal disc. Each parameter was varied independently, while all others were fixed at their previously determined optima. Once the optimum value of one parameter was obtained, it was fixed and used in the next experiment to optimize the next parameter. Although a single factor was used for simplicity, we acknowledge that interaction effects were not assessed and may influence the best-performing conditions. Cultures were incubated at 28 °C for 7 days. In each optimizing experiment, biomass was harvested by filtration and quantified gravimetrically [22]. Total lipids were extracted and quantified by the Folch gravimetric method [24,27]. Growth conditions, including incubation period (1–12 days), initial pH (1–9), temperature (10–45 °C), and agitation (200 rpm shaking versus static culture) at 28 °C, were evaluated for their effects on fungal growth and lipid production. Nutritional preliminary screening was performed in Czapek-Dox broth. Initially, different C sources, including glucose, sucrose, maltose, starch, and cellulose, were individually tested at 30 g L^−1^. Similarly, salinity effects were tested with 5% and 10% (*w*/*v*) NaCl. This stepwise preliminary screening identified the conditions that gave the highest FAME in *F. solani* 102F.

### 2.6. Preliminary Screening of C and N Combination for Enhanced Lipid Accumulation

After selecting the best C and N sources from the single-factor experiments, we tested ten different C and N combinations to assess their influence on C and N balance and lipid accumulation. Sucrose and glucose were used at 30 g L^−1^ as selected C sources, and sodium nitrate, peptone, yeast extract, casein, and ammonium sulfate were individually used at 1.5 g L^−1^ as N sources. All other culture conditions were kept at the previously suitable levels.

### 2.7. Direct Transesterification of Lipid from Fungal Biomass

FAMEs were directly produced from dried fungal biomass using a modified one-step transesterification method [28]. Briefly, 500 mg of dried biomass was suspended in 50 mL of a methanol–hydrochloric acid–chloroform mixture (10:1:1, *v*/*v*/*v*) and heated at 90 °C for 1 h in a thermostatic water bath. Following the reaction, the esterified products were extracted with a *n*-hexane–chloroform mixture (4:1, *v*/*v*), dried over anhydrous sodium sulfate, and separated using a separating funnel. The FAME-containing fraction was collected, evaporated to dryness, and weighed. Biodiesel yield was calculated based on the dry biomass weight using the following equation:

Biodiesel yield % = (weight of FAMEs/weight of dry biomass) × 100

### 2.8. Transesterification of Extracted Fungal Lipids Using Eggshell-Derived CaO

For heterogeneous catalysis, a waste eggshell (ES)-derived CaO catalyst was prepared with minor modifications based on the procedure reported by Dalvand and Mahdavian [29] with minor modifications. ES waste was washed three times with ethanol and distilled water, dried at 100 °C for 24 h, pulverized, and calcined at 900 °C for 4 h in a muffle furnace to obtain CaO.

The extracted fungal lipid/oil fraction was subsequently subjected to transesterification using the ES-derived CaO catalyst following a modified procedure of Borah et al. [30]. The extracted lipid/oil fraction was reacted with methanol at a 20:1 molar ratio in the presence of the ES-derived CaO catalyst at a concentration of 1 gm relative to the oil in a 100 mL three-necked round-bottom flask equipped with a reflux condenser, magnetic stirrer, and thermometer. The mixture was stirred at 600 rpm and maintained at 65 °C for 4 h using a constant-temperature water bath. After reaction completion, the mixture was transferred to a separating funnel to allow phase separation. The biodiesel (FAME) fraction was collected and purified, while the glycerol phase was separated.

### 2.9. GC-Based FAME Profiling

The FAMEs, obtained via direct transesterification and ES-catalyzed reactions, were dissolved in chloroform and analyzed by GC–MS. Analyses were performed using a Trace GC1310-ISQ mass spectrometer (Thermo Scientific, Austin, TX, USA) at the Regional Center for Mycology and Biotechnology (RCMB), Al- Azhar University, with a direct capillary column TG-5MS (30 m × 0.25 mm × 0.25 µm film thickness). Data processing was conducted using AMDIS for spectral deconvolution and compound identification. One microliter of sample was injected with helium as the carrier gas at a flow rate of 1.5 mL min^−1^. The oven temperature program was as follows: 45 °C for 1 min, ramped to 280 °C at 3 °C min^−1^. Injector and mass transfer line temperatures were set at 250 °C and 200 °C, respectively. FAME components were identified by comparing retention times and mass spectra with the NIST 11 mass spectral library (Wiley Registry, mainlib, and replib) [31,32]. FAME composition was expressed as relative abundance based on peak area normalization.

### 2.10. Statistical Analysis

All experiments were conducted using three independent biological replicates (*n* = 3), with data presented as means ± SD. *p*-values and F-statistics are provided in Tables S1 and S2. One-way analysis of variance (ANOVA), followed by Tukey’s post-hoc test, was used to evaluate differences in biomass, lipid content, and yield (SPSS Statistics v. 31.0). *FAS1* gene expression was analyzed by a two-tailed Student’s *t*-test. The catalytic efficiency of ES-derived CaO was compared by one-way ANOVA with Tukey’s post-hoc test. Heatmaps and correlation matrices were generated using the ‘pheatmap’ and ‘corrplot’ packages in R.v.3.4.5 with Figures prepared using ggplot2. To evaluate the joint effect of C source and N source on lipid concentration and lipid yield, and to formally test for a C × N interaction, data from the ten C–N combinations (*n* = 3) were analyzed by two-way ANOVA with C, N, and their C × N interaction as fixed factors, using the car package. Normality of residuals was assessed by the Shapiro–Wilk test and homogeneity of variance by Levene’s test. Where the interaction term was significant, pairwise comparisons among the ten C × N combinations were performed using Tukey’s HSD post-hoc test on the combined factor.

## 3. Results

### 3.1. Fungal Screening and Identification for Lipid Production

**Figure 1:**
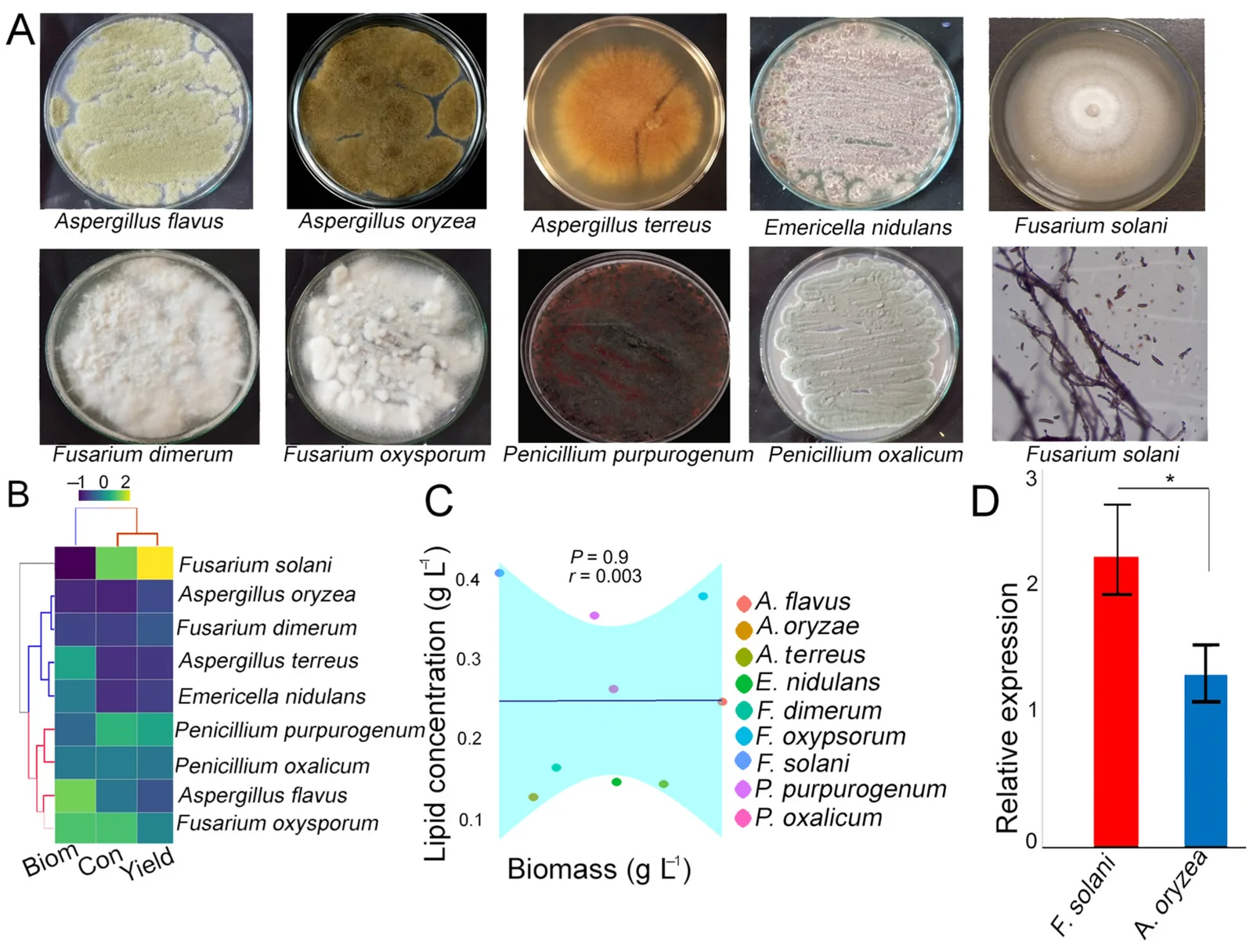
Morphological diversity and quantitative analysis of biomass and lipid production across fungal isolates. (**A**) Representative colony morphologies of *Aspergillus flavus*, *Aspergillus oryzae*, *Aspergillus terreus*, *Emericella nidulans*, *Fusarium dimerum*, *Fusarium oxysporum*, *Fusarium solani*, *Penicillium oxalicum* and *Penicillium purpurogenum*. (**B**) Hierarchical clustering heatmap shows normalized biomass, lipid concentration, and lipid yield across different fungal species. Color bar indicates high (yellow) and low (blue) biomass and lipid production. (**C**) Correlation analysis between biomass and lipid concentration across isolates using Pearson correlation. (**D**) Relative expression of *Fatty acid synthase* (*FAS*) gene in *F. solani* 102 F. and *A. oryzae*, using *Tubulin* as a reference gene. Asterisks denote significant differences (*p* < 0.05) using Student’s *t*-test.

**Figure 2:**
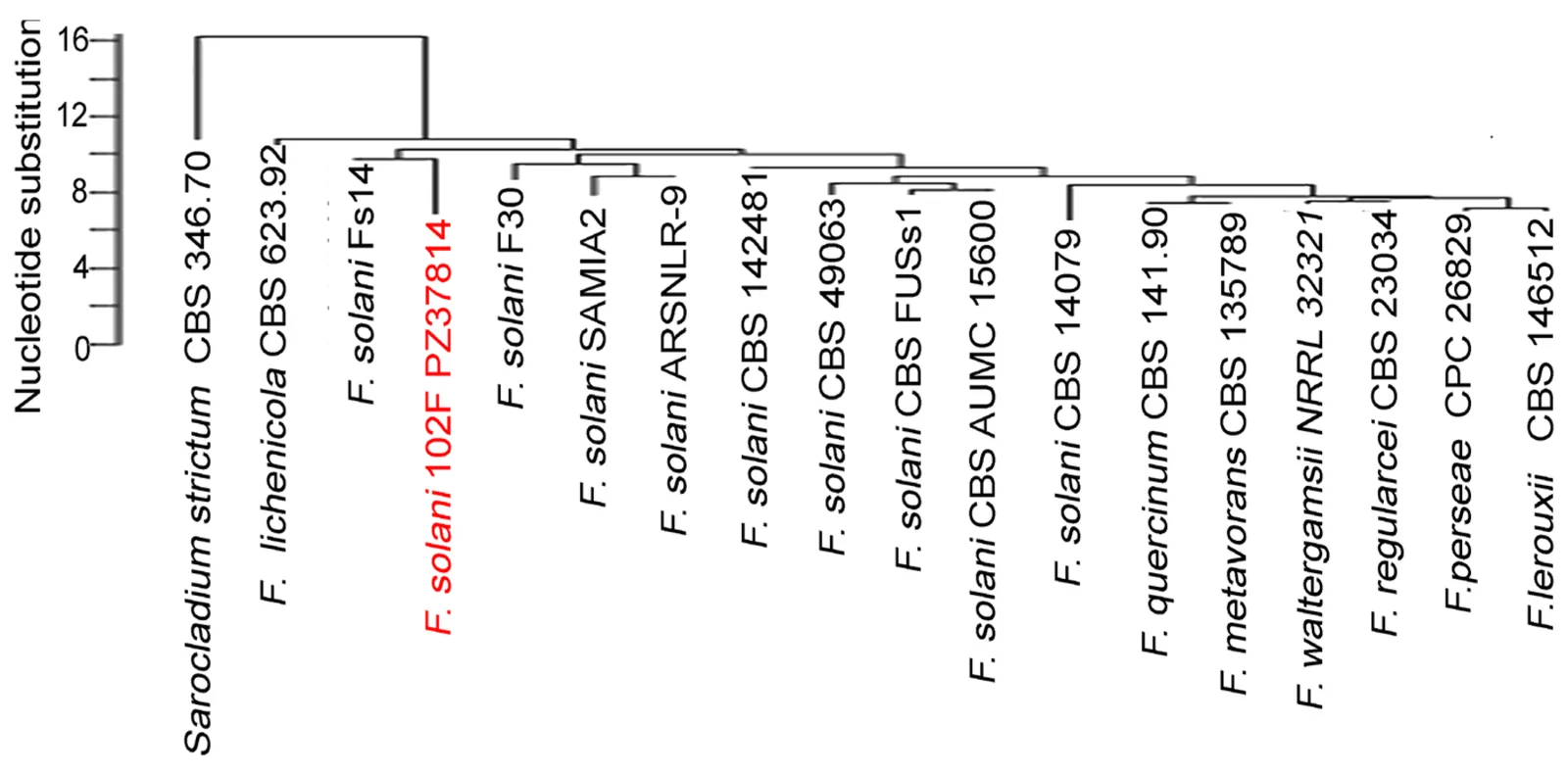
Phylogenetic tree based on ITS rDNA sequences showing the placement of *F. solani* isolate 102F (PZ374814) among closely related taxa retrieved from GenBank.

Endophytic fungi were isolated from *D. sissoo*, a drought-tolerant legume tree common in the arid regions of Aswan, Egypt. These isolates were screened for their ability to produce lipids. Morphological and cultural analyses revealed nine oleaginous fungal species across three genera, including *Aspergillus* (*A. flavus*, *A. oryzae*, *A. terreus*, and *Emericella nidulans*), *Fusarium* (*F. dimerum*, *F. oxysporum*, and *F. solani*), and *Penicillium* (*P. oxalicum* and *P. purpurogenum*) (Figure 1A). Quantitative analysis of biomass and lipid production showed clear differences among the isolates (Figure 1B, Table S1). *F. solani* produced the highest lipid concentration of 0.46 g L^−1^, and 17.1% yield even though its biomass was relatively low. In contrast, *A. oryzae* gave the lowest lipid concentration of 0.13 g L^−1^ and 4.1% yield (Figure 1B, Table S1). *A. flavus* produced the highest biomass 5.64 g L^−1^ but moderate lipid concentration of 0.27 g L^−1^ and 4.8% yield (Figure 1B, Table S1). These observed differences between biomass accumulation and lipid productivity were further supported by a non-significant Pearson correlation (*r* = 0.003) (Figure 1C). To explore the genetic basis of this inter-species variation, we measured the relative expression level of the *FAS1* gene, a key marker for lipid biosynthesis, in the high-lipid producer *F. solani* and the low-lipid producer *A. oryzae* (Figure 1D). *FAS1* transcript levels, measured relative to *β-tubulin*, were higher in *F. solani* 102F than in *A. oryzae* under the tested conditions. However, as primer amplification efficiency and specificity were not independently validated across the two species, this comparison should be interpreted as preliminary rather than a confirmed quantitative difference in *FAS1* transcription. Based on its morphological and cultural features, the highest lipid-yielding *F. solani* was selected for further characterization (Figure 1D). Phylogenetic analysis of the ITS region of rDNA supported its taxonomic placement within the *F. solani* species group (Figure 2). The 603 bp ITS sequence (GenBank accession number PZ374814) showed 94.01–97.27% identity to the *F. solani* Fs14 strain retrieved from GenBank. Sequence alignment with closely related *Fusarium* strains supported this relationship. A maximum-likelihood phylogeny positioned our strain within the *Fusarium* clade, showing the closest relationship with *F. solani* Fs14, while *Sarocladium strictum* was used as an outgroup (Figure 2). This isolate was designated *F. solani* 102F.

### 3.2. Preliminary Screening of Lipid Production by F. solani 102F

**Figure 3:**
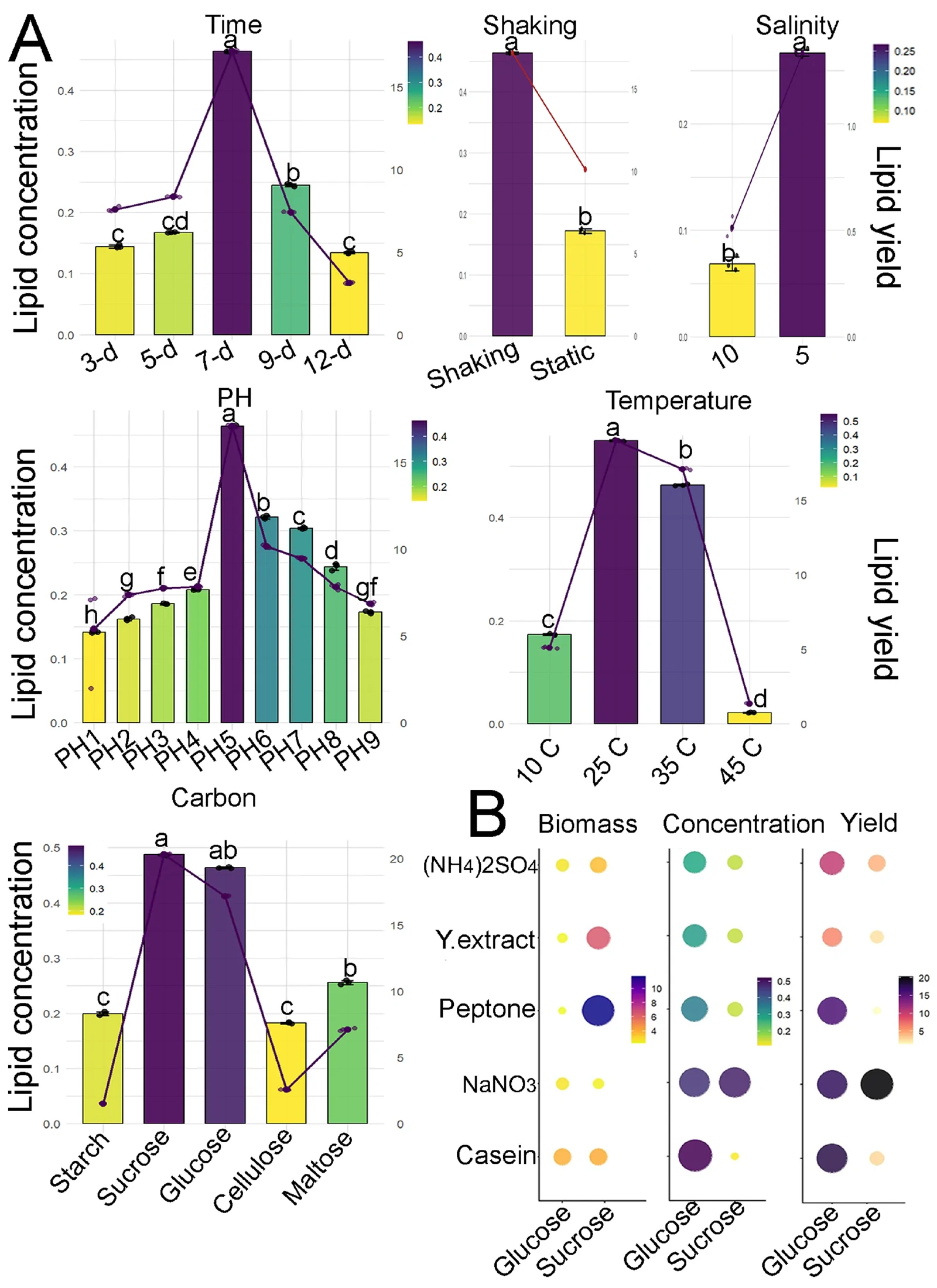
Preliminary screening of culture conditions for lipid production in *Fusarium solani* 102F. (**A**) Bar plots of lipid concentration (g L^−1^, left y-axis) and line plots of lipid yield (%, right y-axis) under various cultivation parameters, including incubation time (3–12 days), agitation (shaking vs. static), salinity (5–10 g L^−1^), pH (pH 1–9), carbon sources (starch, sucrose, glucose, cellulose, and maltose), and temperature (10–45 °C). Different letters above bars indicate statistically significant differences *p* < 0.05 among investigated parameters using Tukey’s HSD test. (**B**) Bubble plot showing biomass, lipid concentration, and lipid yield for different combinations of carbon and nitrogen sources. Bubble size and color indicate the value of each parameter. Different alphabetical letters indicate significant differences among investigated parameters using Tukey’s HSD test (*p* < 0.05).

Preliminary screening of lipid production by *F. solani* 102F was performed using a one-factor approach. Incubation time was first evaluated as the foundational parameter, after which the suitable duration was fixed for all subsequent experiments. This sequential strategy allowed systematic identification of the most favorable conditions for biomass growth and lipid accumulation (Figure 3A,B). In the early growth phase (day 3), biomass reached 1.9 g L^−1^ with a lipid concentration of 0.14 g L^−1^ (Figure 3A, Table S1). Values increased progressively through the period and peak performance was achieved on day 7, with biomass of 2.7 g L^−1^ and lipid concentration of 0.46 g L^−1^, (Figure 3A, Table S1). After day 7, although biomass continued to increase modestly, lipid concentration and content declined (Figure 3A, Table S1). Therefore, an incubation period of 7 days was selected as the fixed duration for all subsequent preliminary screening experiments.

Using the fixed 7-day incubation period, the effect of agitation was then evaluated by comparing shaking (200 rpm) with non-shaking conditions (Figure 3A). Shaking significantly improved both biomass and lipid production in *F. solani* 102F, providing 2.7 g L^−1^ biomass and 0.46 g L^−1^ lipid concentration (Figure 3A, Table S1). In contrast, non-shaking conditions showed substantially lower values of 1.7 g L^−1^ biomass and 0.17 g L^−1^ lipid concentration (Figure 3A, Table S1). These results indicated that *F. solani* 102F prefers shaking conditions which resulted in greater growth and lipid accumulation rather than static conditions.

Using the previously determined conditions (7-day incubation and shaking at 200 rpm), the effect of initial medium pH on fungal growth and lipid production was then evaluated across a broad range (Figure 3A). The maximum 3.21 g L^−1^ biomass and 0.46 g L^−1^ lipid concentration were obtained at pH 5.0 (Figure 3A, Table S1). In general, stronger acidic pH values (1.0–4.0) reduced both biomass and lipid production compared with slightly acidic to neutral pH (5–7) levels (Figure 3A, Table S1). These results demonstrate that *F. solani* 102F exhibits a clear preference for mildly acidic conditions, with pH 5.0 being the best-performing pH for both growth and lipid biosynthesis.

Next, under the best-performing conditions of 7-day incubation, shaking at 200 rpm, and pH 5.0, the effect of temperature was evaluated at 10, 25, 30, and 45 °C (Figure 3A, Table S1). Results indicated that 25 °C was the best-performing temperature, supporting the highest biomass of 2.89 g L^−1^ and lipid concentration of 0.549 g L^−1^. Performance was markedly lower at 10 °C and declined sharply at 45 °C, where lipid concentration dropped to 0.022 g L^−1^. Therefore, the incubation temperature was fixed at 25 °C for all remaining experiments.

We then used the best-performing conditions of 7-day incubation, shaking at 200 rpm, pH 5.0, and 25 °C to examine the effect of C source on biomass and lipid production by testing five different C sources (starch, sucrose, glucose, cellulose, and maltose) at a concentration of 30 g L^−1^ (Figure 3A). Sucrose and glucose supported the highest lipid concentrations (0.48 and 0.46 g L^−1^, respectively) with the lowest biomass values of 2.4 and 2.7 g L^−1^, respectively (Figure 3A, Table S1). In contrast, starch and cellulose promoted significantly higher biomass production (13.1 g L^−1^ and 7.1 g L^−1^, respectively); however, these two C sources resulted in substantially lower lipid concentrations (0.19 g L^−1^ and 0.18 g L^−1^, respectively) (Figure 3A, Table S1). Collectively, the culture conditions for maximum lipid production by *F. solani* 102F were determined to be: 7 days of incubation at 25 °C with shaking at 200 rpm, initial pH 5.0, and 30 g L^−1^ sucrose.

### 3.3. Effects of Salinity and C/N Combinations on F. solani 102F Growth and Lipid Production

Under the previously determined medium and culture conditions (7 days of incubation at 25 °C with shaking at 200 rpm, initial pH 5.0, and 30 g L^−1^ sucrose), we tested the effect of salinity by adding different concentrations of NaCl to the medium (0, 5, and 10 g L^−1^) (Figure 3A). Salt concentration at 5 g L^−1^ NaCl significantly enhanced both biomass and lipid production (Figure 3A, Table S1). In contrast, increasing the NaCl concentration to 10 g L^−1^ exerted a clear inhibitory effect, resulting in reduced biomass, lipid concentration, and overall FAME (Figure 3A, Table S1). These findings indicate that *F. solani* 102F exhibits improved lipid accumulation under mild salt stress.

A two-way ANOVA confirmed statistically significant main effects of C source and N source on lipid concentration, as well as a significant C × N combination, confirming that the effect of C source on lipid concentration depended on the co-supplied N source (Table S5). The same pattern was observed for lipid yield. As shown previously (Figure 3A, Table S1), sucrose and glucose proved to be the most suitable substrates for lipid accumulation in *F. solani* 102F among the five tested C sources. To identify the best-performing C and N combination, glucose and sucrose (30 g L^−1^) were tested as C sources in combination with five different nitrogen sources (1.5 g L^−1^) under the previously determined growth conditions (7 days of incubation at 25 °C with shaking at 200 rpm, initial pH 5.0, 5 g L^−1^ NaCl and 30 g L^−1^ sucrose or glucose) (Figure 3B). The results revealed a clear difference in lipid production among the tested C/N combinations. Specifically, glucose combined with casein was the best C/N combination, producing the highest lipid concentration of 0.51 g L^−1^ with a biomass of 3.9 g L^−1^ corresponding to a lipid yield of approximately 13.33% (Figure 3B, Table S2). Sucrose combined with sodium nitrate was the second-best combination, yielding a lipid concentration of 0.48 g L^−1^ and 2.40 g L^−1^ biomass but resulted in a higher lipid yield of approximately 20.0% (Figure 3B, Table S2). However, sucrose paired with casein resulted in lower lipid concentration (0.11 g L^−1^) with a biomass of 4 g L^−1^ and lipid yield 2.84%. On the other hand, sucrose paired with peptone supported the highest biomass (11.71 g L^−1^) but poor lipid concentration (0.15 g L^−1^) and lipid yield (1.28%) (Figure 3B, Table S2). In contrast, the combinations with yeast extract, ammonium sulfate, or peptone generally produced lower lipid titers regardless of the C source. Thus, the most effective combination for maximum lipid production was glucose as the C source and casein as the N source. Nevertheless, sucrose and sodium nitrate combination also provided a relatively comparable lipid content (Figure 3B, Table S2). Thus glucose–casein pairing was selected as the best-performing C/N regime for *F. solani* 102F lipid production.

### 3.4. Direct Transesterification of Lipid from Fungal Biomass

*F. solani* 102F was cultivated under the selected conditions (7 days of incubation at 25 °C, 200 rpm, initial pH 5.0, 5 g L^−1^ NaCl, 30 g L^−1^ glucose, and 1.5 g L^−1^ casein), and the resulting dried fungal biomass was subjected to direct transesterification using a one-step procedure. Direct conversion of the dried biomass resulted in the recovery of fatty acid methyl esters (FAMEs), with a FAME yield of 15.8%. This result demonstrated the potential of the fungal biomass as a feedstock for biodiesel production. The recovered FAME fraction was subsequently characterized to determine its fatty acid composition.

### 3.5. Biodiesel Production via Transesterification of Extracted Fungal Lipids Using Eggshell-Derived Cao

**Figure 4:**
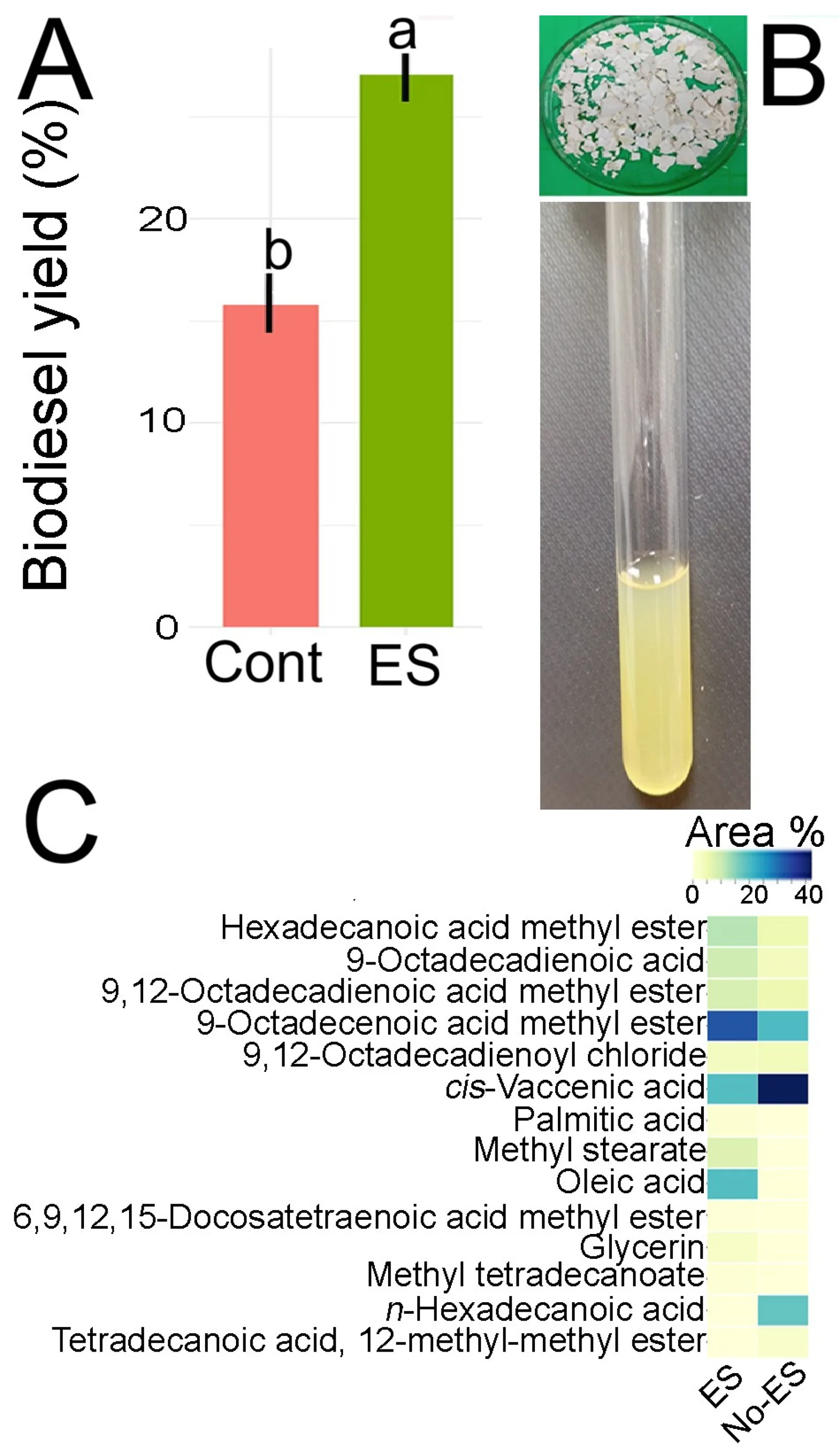
Eggshell (ES)-derived CaO catalyzed biodiesel production from fungal lipids. (**A**) Bar plots of biodiesel yield % in the presence or absence of ES-derived CaO catalyst. Different letters above bars indicate statistically significant differences *p* < 0.05 among investigated parameters using Tukey’s HSD test. (**B**) Representative images of the ES (**top**) and the resulting biodiesel phase after transesterification (**bottom**), illustrating successful lipid conversion. (**C**) Heatmap of fatty acid methyl ester profile with and without ES-assisted transesterification. Color bar indicates relative abundance (area %).

The lipid extracted from *F. solani* 102F grown under determined conditions (7 days of incubation at 25 °C with shaking at 200 rpm, initial pH 5.0, 5 g L^−1^ NaCl, 30 g L^−1^ glucose and 1.5 g L^−1^ casein) was subjected to transesterification using ES-derived CaO catalyst for biodiesel production. ES powder was selected as a waste-derived, calcium-rich precursor for CaO preparation, providing an opportunity for eggshell waste valorization in heterogeneous catalysis [17]. The transesterification reaction was carried out using 0.5 g of microbial lipid with methanol in the presence or absence of ES-derived CaO (1.0 g) as a catalyst. The addition of ES-derived CaO markedly enhanced FAME production compared with the reaction performed without the catalyst (Figure 4A,B, Table S3). The highest FAME yield reached 27.02% in the presence of ES-derived CaO, compared with 15.8% in its absence (Figure 4A,B, Table S3). Based on the lipid used as the substrate for transesterification, this corresponds to a relative increase of approximately 71% in FAME yield following the addition of ES-derived CaO. Because the transesterification experiments were conducted using the extracted microbial lipid fraction rather than the total fungal biomass, these values represent lipid-based FAME yields and should not be interpreted as overall biodiesel yields relative to the initial biomass. These findings indicate that ES-derived CaO can serve as an effective and inexpensive catalyst for the transesterification of *F. solani* 102F microbial lipid.

### 3.6. Lipid Profile Analysis of Fusarium solani 102F by GC–MS

To evaluate the quality of fungal lipids for biodiesel production, we profiled FAMEs using GC–MS. This analysis provides a quantitative framework to evaluate chain length distribution and degree of unsaturation, key determinants of fuel properties. The FAMEs resulting from transesterification with and without ES-derived CaO were analyzed by GC–MS, revealing a diverse composition of fatty acids and their derivatives (Table S4). GC–MS analysis of FAMEs generated by ES-catalyzed transesterification revealed a lipid profile dominated by C18 monounsaturated fatty acids (Figure 4C, Table S4). The major constituents were 9-octadecenoic acid methyl ester with 32.7%, followed by oleic acid and *cis*-vaccenic acid with 19.6% each, indicating a strong enrichment of monounsaturated species (Figure 4C, Table S4). Additional components included hexadecanoic acid methyl ester with 11.65%, 9-octadecenoic acid with 9.47%, and 9,12-octadecadienoic acid methyl ester with 8.4%, while minor fractions of methyl stearate, glycerol, and palmitic acid were also detected (Figure 4C, Table S4). In contrast, transesterification without ES-derived catalyst yielded a distinct lipid distribution, with *cis*-vaccenic acid as the predominant component with 42.23%, followed by 9-octadecenoic acid methyl ester with 20.12%, and *n*-hexadecanoic acid with 18.08% (Figure 4C, Table S4). Lower-abundance compounds included hexadecanoic acid methyl ester with 5.19%, 9-octadecenoic acid with 3.64%, and 9,12-octadecadienoic acid methyl ester with 4.95%. Notably, oleic and palmitic acids were not detected under transesterification without the ES-derived catalyst. Collectively, ES-derived CaO-associated transesterification of *F. solani*-derived lipids are enriched in C16–C18 fatty acids, with a predominance of mono- and polyunsaturated species.

## 4. Discussion

Oleaginous fungi are becoming increasingly popular for biodiesel production because they are inexpensive and can efficiently produce lipids, often outperforming other microorganisms [33]. Fungi that accumulate lipids also offer a promising alternative source with multiple potential applications [34]. In this study, nine fungal isolates were screened to evaluate their lipid accumulation ability. Several well-known oleaginous filamentous fungi used in biodiesel research, including *A. terreus*, *A. niger*, *F. oxysporum*, and *Mucor circinelloides*, can produce substantial amounts of lipids [35]. Among the isolates tested, *F. solani* 102F produced the highest lipid concentration, whereas *A. oryzae* displayed the lowest lipid production (Figure 1A,B, Table S1). It is worth noting that the high lipid production was not correlated with biomass, indicating a shift in the fungal metabolism for lipid accumulation rather than vegetative growth (Figure 1C). The *FAS1* gene revealed a higher expression level in *F. solani* 102F compared with *A. oryzae* (Figure 1D). These results align well with the documented capacity of *Fusarium* spp. as high-yield lipid producers [22,36].

Culture conditions and nutrient availability play a key role in boosting microbial lipid productivity and yield during fermentation [37]. Lipid content can vary significantly depending on the microorganism itself, as well as several culture factors, including incubation time, temperature, medium pH, type of C and N sources, and whether the cultures are kept static or agitated [37,38]. In this study, *F. solani* 102F showed a gradual increase in both biomass and lipid production, with both reaching their maximum on day 7. Thereafter, biomass continued rising slightly, while lipid concentration began to decline (Figure 3A, Table S1). The reduction in lipid accumulation during the later stationary phase is likely attributable to nutrient depletion and/or accumulation of inhibitory metabolites [39]. This decline may be attributed to the onset of lipid turnover, where stored TAGs are metabolized as an endogenous C source to sustain maintenance and cellular activities once essential nutrients become exhausted [22,40].

A comparative experiment between static and shaking (200 rpm) conditions showed that shaking significantly enhanced both biomass and lipid production in *F. solani* 102F (Figure 3A, Table S1). This improvement is likely due to better oxygen transfer, which in turn supports better cell growth and metabolic activity [41,42]. Similar positive effects of shaking on lipid accumulation were also observed in *F. verticillioides* [36]. The pH of the growth medium was another key factor affecting biomass production and lipid accumulation. This is because the concentration of hydrogen ions influences plasma membrane permeability and the transport of substances in and out of the cells [43]. Under the sequential OFAT screening strategy, pH 5.0 resulted in the highest lipid production, while the highest biomass was obtained at pH 6.0 (Figure 3A, Table S1). Lipid production dropped sharply under strongly acidic or alkaline conditions. These findings agree with those reported by Abdellah et al. [44], which showed that pH 5.0 was best for lipid production by *Aspergillus* sp. strain EM2018. However, other oleaginous fungi have shown maximum lipid production at different pH levels, such as pH 6 or 7 [22,23].

Temperature had a strong effect on biomass formation and lipid synthesis in the *F. solani* (Figure 3A, Table S1). Under the sequential OFAT screening strategy, 25 °C resulted in the highest fungal biomass and lipid accumulation (Figure 3A). This is likely because this temperature supports the production of extracellular enzymes involved in breaking down polysaccharides and promoting lipid storage [45]. Additionally, the C source plays a major role in regulating lipid biosynthesis while supporting biomass formation, as acetyl-CoA is primarily derived from pyruvate, the product of glycolysis [46,47]. To optimize lipid accumulation and biomass production in *F. solani* 102F, different C sources were then tested (Figure 3A, Table S1). Among the C sources evaluated, sucrose and glucose were the most effective for lipid production (Figure 3A, Table S1). These findings agree with previous reports showing that various C sources can support lipid accumulation in oleaginous microorganisms [48]. Glucose is widely considered one of the best C sources for TAG production because it is readily available, inexpensive, and easily metabolized by microbial cells without the need for additional enzymes [47,49]. Similar results have been reported in several studies, where glucose supported the highest lipid yields in oleaginous fungi [47,49,50]. In addition, sucrose also gave high lipid production in this study, which aligns with those reported by Wadee et al. [27], who observed maximum biomass and lipid yields in *F*. *verticillioides* AUMC when using sucrose. Salinity is a potent stressor that maximizes intracellular lipid accumulation in oleaginous fungi. Fungi accumulate TAGs under salt stress primarily as a survival strategy, redirecting excess carbon into energy-dense lipid storage while protecting cells from oxidative stress and maintaining membrane integrity [3]. In our study, moderate salt stress significantly increased both biomass and lipid production in *F. solani* 102F (Figure 3A, Table S1). However, higher NaCl concentrations had a clear inhibitory effect, leading to reduced biomass, lipid concentration, and overall FAME (Figure 3A, Table S1). These findings suggest that *F. solani* 102F can improve lipid accumulation under mild osmotic stress, possibly by activating stress-responsive mechanisms [51]. The present study represents a preliminary screening of the major culture parameters using a sequential OFAT approach. Further process optimization using multivariable statistical approaches, such as Response Surface Methodology (RSM), would be required to evaluate interactions among variables and establish statistically optimized conditions.

The N source is also an essential component of microbial growth media, strongly influencing not only growth but also the production of bioactive compounds and lipid accumulation in oleaginous microorganisms [52]. In nitrogen-limiting conditions, high glucose concentrations are proposed to enhance carbon flux towards triacylglycerol (TAG) production, which is then sequestered in liposomes, hence promoting lipid accumulation in many organisms [53]. The present findings also demonstrate that the C and N combination significantly influenced lipid biosynthesis in *F. solani* 102F (Figure 3B). Interestingly, glucose combined with casein was the best C and N combination, producing the highest lipid concentration of 0.51 g L^−1^ with a biomass of 3.9 g L^−1^ (Figure 3B, Table S2). This enhancement may be attributed to the efficient utilization of glucose as a readily metabolizable C source that rapidly enters glycolytic pathways, providing sufficient acetyl-CoA and reducing equivalents required for lipid biosynthesis.

Simultaneously, casein serves as an organic N source rich in amino acids and peptides that support fungal growth, enzymatic activity, and cellular metabolism, thereby promoting biomass formation and subsequent lipid accumulation. The balance between an easily assimilated C source and N supply is thought to have a major role in controlling the synthesis of TAG and oleaginous metabolism in fungi [47]. Similarly, Robles-Iglesias et al. [54] reported that the interaction between C and N sources strongly regulates lipid accumulation in oleaginous microorganisms, emphasizing that optimized C and N combinations are more important than individual nutrient effects alone.

In-situ transesterification offers a promising alternative to conventional extraction and transesterification methods for fungal lipids. This approach allows lipids to be extracted and converted into esters in a single step, which can significantly reduce processing costs. The traditional method typically uses alcohol with a homogeneous acid catalyst, such as sulfuric or hydrochloric acid, to convert free fatty acids into esters [55]. Producing biodiesel from microbial lipids using catalysts derived from waste materials represents a sustainable route for renewable energy, which also supports waste management and environmental protection [56]. Catalysts play a critical role in biodiesel production cost, and the use of waste-derived materials can substantially improve the economics of the process [57]. In this study, lipids extracted from *F. solani* 102F grown under determined conditions were converted into biodiesel via transesterification using ES powder as a low-cost, eco-friendly, heterogeneous catalyst. ES are rich in CaCO_3_ and can be easily converted into an active CaO catalyst through high-temperature calcination [30]. Compared with commercial CaCO_3_, ES-derived CaO shows unique performance at high temperatures, as well as good adsorption capacity for organic molecules, and helps in the easy separation of ethyl esters from glycerol, often eliminating the need for additional purification steps [29]. Our results showed that the ES-derived catalyst produced a significantly higher biodiesel yield (27.02%) than non-ES transesterification (15.8%) (Figure 4A, Table S3), representing a 71% increase (Figure 4A, Table S3). This finding indicates that the calcined ES-derived material enhanced the conversion of *F. solani* 102F microbial oil to FAMEs under the reaction conditions employed. However, the absolute yield obtained in the present study was lower than that reported by Yaşar [58], who achieved a maximum biodiesel yield of 95.12% using calcined waste ES as a catalyst. Therefore, the obtained 27.02% yield should be considered a preliminary indication of the catalytic potential of the ES-derived material rather than an optimized biodiesel yield. Although eggshell-derived CaO increased the FAME yield from 15.8% to 27.02%, the absolute yield was lower than those reported for some waste-derived CaO catalysts. This may be partly due to the lack of systematic optimization of the reaction conditions, including the 20:1 methanol-to-oil ratio, catalyst loading, reaction time, and temperature. Therefore, the obtained yield represents the catalytic performance under the tested conditions rather than the maximum achievable conversion. Further optimization and process evaluation are required to assess its potential for industrial scale-up. Overall, these findings demonstrate that ES powder is an effective and inexpensive catalyst for the transesterification of *F. solani* 102F microbial oil.

The increasing interest in renewable and non-edible lipid feedstocks has encouraged the development of alternative biodiesel production routes, with transesterification remaining a key step for converting lipids into fatty acid methyl esters (FAMEs). Recent studies have emphasized the importance of feedstock selection, conversion technology, and process sustainability in developing viable biodiesel production systems [59]. Recent work has demonstrated the value of multivariable statistical optimization in improving biodiesel production; for example, Ao et al. [60] applied Response Surface Methodology (RSM) to optimize biodiesel production using a biomass-derived carbon catalyst and achieved a biodiesel yield of 97.1 ± 0.4% under the optimized conditions. These findings further support the need for systematic optimization of catalyst loading, methanol-to-oil ratio, temperature, and reaction time in future studies to improve the FAME yield obtained from *F. solani* 102F lipid.

In the present study, catalyst recovery and reuse over successive transesterification cycles were not experimentally evaluated. Therefore, although previous studies in the literature support the potential recyclability of eggshell-derived CaO, the stability and number of effective reuse cycles of the prepared catalyst cannot be established from the present data. Heterogeneous CaO catalysts offer the important advantage of potential recovery and reuse after transesterification, which can reduce catalyst consumption and operating costs. Previous studies have demonstrated the potential reusability of waste eggshell-derived CaO catalysts for biodiesel production. Khemthong et al. [61] reported that eggshell-derived CaO could be recovered and reused in successive biodiesel production cycles, while Niju et al. [62] demonstrated the suitability of waste eggshell-derived CaO as a heterogeneous catalyst in a continuous-flow process. However, the catalytic stability of CaO during repeated use may be affected by several factors, including leaching of active Ca species, carbonation and hydration of CaO, and changes in the catalyst surface and basic active sites [63]. More recently, Macheli et al. [64] reported a gradual decrease in the activity of unmodified waste eggshell-derived CaO during repeated use, highlighting the potential for catalyst deactivation and the importance of improving catalyst stability. Further studies should investigate catalyst recovery, activity retention, and structural stability over multiple reaction cycles to determine its practical reusability and suitability for larger-scale biodiesel production.

GC–MS-based FAME profiling is regarded as one of the most dependable analytical methods for assessing the biodiesel potential of oleaginous microorganisms because it allows for the accurate identification and quantification of fatty acids that directly affect fuel characteristics [65]. Our study investigated that transesterified FAMEs with and without ES-catalyzed reactions showed diverse compositions of fatty acids and their derivatives (Figure 4B). GC–MS analysis of FAMEs generated by ES-catalyzed transesterification revealed a lipid profile dominated by C18 monounsaturated fatty acids (Figure 4C, Table S4). This agreed with results of Michael and Baskaran [66], who reported that fungal lipids high in palmitic acid (C16:0), stearic acid (C18:0), oleic acid (C18:1), and linoleic acid (C18:2) are especially favorable for biodiesel generation. High oleic acid content specifically enhances oxidative stability and ignition quality [67]. As a result, FAME composition is a crucial metric for evaluating microbial biodiesel’s fuel efficiency and industrial viability. ES-derived catalysts greatly increase transesterification efficiency and biodiesel yield due to their high catalytic activity, porous surface structure, and potent alkaline characteristics. Additionally, because ES-derived catalysts are reusable and simple to separate from the reaction mixture, they offer an economical and eco-friendly substitute for traditional chemical catalysts [68,69].

## 5. Conclusions

This study demonstrates that the endophytic fungus strain *F. solani* 102F, isolated from a drought-adapted legume host, is a promising microbial platform for sustainable biodiesel production. Through stepwise preliminary screening of medium and culture conditions, we achieved substantial improvements in biomass and lipid accumulation, with the highest yields obtained using glucose and casein as C and N sources, respectively, under 7-day incubation, 200 rpm shaking, pH 5.0, and 25 °C. Moderate salinity stress enhanced lipid productivity, revealing the strain’s resilience and metabolic flexibility under controlled stress. Direct transesterification of *F. solani* 102F biomass demonstrated its potential as a microbial feedstock for biodiesel production. Importantly, we showed that low-cost waste ES-derived CaO serves as an effective heterogeneous catalyst for the transesterification of extracted fungal lipids, markedly increasing biodiesel yield while reducing processing costs. The resulting FAME profile, dominated by C16–C18 fatty acids with high monounsaturated content, meets key requirements for efficient biodiesel production. Taken together, our findings demonstrate an integrated fungi-to-fuel approach that combines fungal lipid production with the utilization of an eggshell waste-derived CaO catalyst for biodiesel production. This strategy highlights the potential for converting fungal lipids into renewable fuel while contributing to the valorization of eggshell waste within a circular bioeconomy framework. Future work scaling up this process and assessing its life-cycle impacts could accelerate the development of truly sustainable alternatives to fossil diesel.

## Data Availability

The data supporting the findings of this study are available within the article and its Supplementary Materials.

## Supplementary Materials

The following supporting information can be downloaded at: https://www.mdpi.com/article/10.3390/microorganisms14102272/s1. Table S1. Diversity analysis of biomass production, lipid concentration, and lipid yield across nine fungal isolates, as well as stepwise optimization of culture conditions for lipid production in *Fusarium solani* 102F. (A) A comparative screening of nine fungal isolates under identical baseline conditions. (B) Sequential single-factor optimization for lipid production in *Fusarium solani* 102F, including incubation time, agitation mode, salinity, initial pH, nitrogen source, carbon source, temperature, and trace element concentrations. Values are mean ± SD (*n* = 3). One-way ANOVA was performed for each investigated parameter. Table S2. Effects of carbon and nitrogen combinations on biomass production, lipid concentration, and lipid yield in *Fusarium solani* 102F. Values are mean ± SD (*n* = 3). Table S3. Biodiesel production via direct transesterification using eggshell as a heterogeneous catalyst in *Fusarium solani* 102F. Values are mean ± SD (*n* = 3). * Best-performing combination with highest biodiesel yield. Table S4. Gas chromatography mass spectrometry (GC–MS) profiling of fatty acid methyl ester composition under direct transesterification and eggshell-catalyzed transesterification of lipids from *Fusarium solani* 102F. Values represent relative peak area (%). RT, retention time. Table S5. Two-way analysis of variance (ANOVA) testing the effects of carbon source, nitrogen source, and their interaction on lipid concentration (g/L) and lipid yield (%) produced by *Fusarium solani* 102F.
