# Supplementary material for: High Lipid Yield and Efficient Biodiesel Production from Fusarium solani 102F Using Waste Eggshell as Biocatalyst
Source: Microorganisms. 2026 Oct 6;14(10):2272. doi: 10.3390/microorganisms14102272 (PMC13707833; doi:10.3390/microorganisms14102272)
Supplement: Supplementary file 1 [file microorganisms-14-02272-s001.zip › microorganisms-4572370-supplementary.pdf]

Table S1. Diversity analysis of biomass production, lipid concentration, and lipid yield across nine fungal isolates, as well as stepwise optimization of culture conditions for lipid production in *Fusarium solani* 102F**.** A) A comparative screening of nine fungal isolates under identical baseline conditions. B) Sequential single-factor optimization for lipid production in *Fusarium solani* 102F, including incubation time, agitation mode, salinity, initial pH, nitrogen source, carbon source, temperature, and trace element concentrations. Values are mean ± SD (n = 3). One-way ANOVA was performed for each investigated parameter.

	**Biomass ($g\ L^{-1}$)**	**Lipid concentration ($g\ L^{-1}$)**	**Lipid yield (%)**
**A. Fungal species screening**			
Penicillium purpurogenum	3.96 ± 0.05	0.39 ± 0.01	10.09 ± 0.07
Aspergillus flavus	5.64 ± 0.06	0.27 ± 0.00	4.81 ± 0.04
Aspergillus terreus	4.87 ± 0.06	0.14 ± 0.01	3.06 ± 0.04
Aspergillus oryzae	3.16 ± 0.06	0.13 ± 0.01	4.10 ± 0.24
Emericella nidulans	4.25 ± 0.06	0.15 ± 0.00	3.58 ± 0.02
Fusarium dimerum	3.46 ± 0.06	0.17 ± 0.01	5.01 ± 0.19
Fusarium solani 102F	2.71 ± 0.06	0.46 ± 0.00	17.10 ± 0.06
Penicillium oxalicum	4.21 ± 0.06	0.28 ± 0.01	6.89 ± 0.11
Fusarium oxysporum	5.39 ± 0.06	0.42 ± 0.01	7.95 ± 0.02
F-statistic	838.11	3367.57	4655.96
Anova p-value	1.54×10^{-21}***	5.79×10^{-27}***	3.14×10^{-28}***
**B. Optimization parameters**			
**Incubation time**			
3 days	1.90 ± 0.06	0.14 ± 0.00	7.59 ± 0.15
5 days	2.00 ± 0.06	0.17 ± 0.00	8.36 ± 0.04
7 days	2.70 ± 0.06	0.46 ± 0.00	17.16 ± 0.06
9 days	3.30 ± 0.06	0.24 ± 0.00	7.40 ± 0.07
12 days	4.30 ± 0.06	0.13 ± 0.00	3.12 ± 0.03
F-statistic	823.33	16295.09	12290.06
Anova p-value	1.52×10^{-12}***	5.10×10^{-19}***	2.09×10^{-18}***
**Agitation**			
Static	1.70 ± 0.06	0.17 ± 0.00	10.11 ± 0.22
Shaking	2.70 ± 0.06	0.46 ± 0.00	17.16 ± 0.06
F-statistic	416.67	16288.73	3040.87
Anova p-value	3.40×10^{-5}***	2.26×10^{-8}***	6.47×10^{-7}***
**Salinity (% NaCl)**			
5%	19.80 ± 0.10	0.27 ± 0.00	1.35 ± 0.02
10%	13.20 ± 0.10	0.07 ± 0.01	0.52 ± 0.05

F-statistic	6534.00	2332.57	776.34
Anova *p*-value	1.40×10^{-7}***	1.10×10^{-6}***	9.87×10^{-6}***
**Initial pH**			
pH 1	2.01 ± 0.06	0.14 ± 0.00	5.40 ± 2.96
pH 2	2.20 ± 0.06	0.16 ± 0.00	7.37 ± 0.11
pH 3	2.40 ± 0.06	0.19 ± 0.00	7.74 ± 0.04
pH 4	2.65 ± 0.06	0.21 ± 0.00	7.84 ± 0.04
pH 5	3.21 ± 0.06	0.46 ± 0.00	17.10 ± 0.06
pH 6	3.19 ± 0.06	0.32 ± 0.00	10.17 ± 0.08
pH 7	3.16 ± 0.06	0.30 ± 0.00	9.48 ± 0.05
pH 8	3.11 ± 0.06	0.24 ± 0.01	7.84 ± 0.16
pH 9	2.52 ± 0.06	0.17 ± 0.00	6.85 ± 0.09
F-statistic	180.04	5468.38	35.06
Anova *p*-value	1.41×10^{-15}***	7.40×10^{-29}***	1.96×10^{-9}***
**Nitrogen source**			
Casein	2.90 ± 0.06	0.52 ± 0.00	17.89 ± 0.03
Peptone	2.20 ± 0.06	0.35 ± 0.00	15.80 ± 0.23
Ammonium sulphate	2.70 ± 0.06	0.28 ± 0.00	10.27 ± 0.04
Yeast extract	4.70 ± 0.06	0.30 ± 0.00	6.32 ± 0.03
$NaNO_3$	2.70 ± 0.06	0.46 ± 0.00	17.16 ± 0.06
F-statistic	773.33	5321.49	6355.50
Anova *p*-value	2.08×10^{-12}***	1.37×10^{-16}***	5.64×10^{-17}***
**Carbon source**			
Starch	13.10 ± 0.10	0.20 ± 0.00	1.52 ± 0.03
Sucrose	2.40 ± 0.06	0.49 ± 0.00	20.30 ± 0.06
Glucose	2.70 ± 0.06	0.46 ± 0.00	17.16 ± 0.06
Cellulose	7.10 ± 0.06	0.18 ± 0.00	2.57 ± 0.02
Maltose	3.60 ± 0.06	0.26 ± 0.00	7.10 ± 0.11
F-statistic	12447	10384	59642
Anova *p*-value	1.96×10^{-18}***	4.85×10^{-18}***	7.76×10^{-22}***
**Temperature (°C)**			
10°C	2.39 ± 0.06	0.17 ± 0.00	5.11 ± 0.05
25°C	2.89 ± 0.06	0.55 ± 0.00	19.02 ± 0.06
30°C	2.70 ± 0.06	0.46 ± 0.00	17.10 ± 0.06
45°C	1.61 ± 0.06	0.02 ± 0.00	1.35 ± 0.05

F-statistic	265.08	89900.33	87015.26
Anova *p*-value	2.41×10^{-8}***	1.90×10^{-18}***	2.17×10^{-18}***
**Trace elements (mM)**			
Cd^{2+} 0.01	2.61 ± 0.06	0.32 ± 0.00	12.24 ± 0.01
Cd^{2+} 0.03	2.11 ± 0.06	0.26 ± 0.00	12.33 ± 0.02
Cd^{2+} 0.05	1.31 ± 0.06	0.17 ± 0.00	12.96 ± 0.11
Zn^{2+} 0.01	2.19 ± 0.06	0.06 ± 0.00	2.78 ± 0.04
Zn^{2+} 0.03	2.17 ± 0.06	0.19 ± 0.00	8.65 ± 0.06
Zn^{2+} 0.05	2.73 ± 0.06	0.35 ± 0.00	12.77 ± 0.06
Pb^{2+} 0.01	3.28 ± 0.06	0.46 ± 0.00	13.93 ± 0.03
Pb^{2+} 0.03	3.76 ± 0.06	0.39 ± 0.00	10.48 ± 0.08
Pb^{2+} 0.05	4.41 ± 0.06	0.34 ± 0.01	7.62 ± 0.13
F-statistic	749.91	7973.03	7432.77
Anova *p*-value	4.19×10^{-21}***	2.49×10^{-30}***	4.68×10^{-30}***

Table S2. Effects of carbon and nitrogen combinations on biomass production, lipid concentration, and lipid yield in *Fusarium solani* 102F. Values are mean ± SD (n = 3).

**Carbon source**	**Nitrogen source**	**Biomass (g L^{-1})**	**Lipid concentration (g L^{-1})**	**Lipid yield (%)**
Glucose	$(NH_4)_2SO_4$	2.70 ± 0.06	0.28 ± 0.00	10.27 ± 0.04
Sucrose	$(NH_4)_2SO_4$	3.60 ± 0.06	0.15 ± 0.00	4.29 ± 0.06
Glucose	Yeast extract	2.30 ± 0.06	0.30 ± 0.00	6.30 ± 0.01
Sucrose	Yeast extract	6.40 ± 0.06	0.15 ± 0.00	2.40 ± 0.03
Glucose	Peptone	2.20 ± 0.06	0.35 ± 0.01	15.80 ± 0.23
Sucrose	Peptone	11.71 ± 0.06	0.15 ± 0.00	1.28 ± 0.01
Glucose	$NaNO_3$	2.70 ± 0.06	0.46 ± 0.00	17.16 ± 0.06
Sucrose	$NaNO_3$	2.40 ± 0.06	0.49 ± 0.00	20.30 ± 0.06
Glucose	Casein	3.90 ± 0.06	0.52 ± 0.00	13.33 ± 0.03
Sucrose	Casein	4.00 ± 0.06	0.11 ± 0.00	2.84 ± 0.06
F-statistic		7136.97	15981.15	23260.99
Anova p-value		4.67×10^{-33}***	1.48×10^{-36}***	3.46×10^{-38}***

Table S3. Biodiesel production via direct transesterification using eggshell as a heterogeneous catalyst in *Fusarium solani* 102F**.** Values are mean ± SD (n = 3). * Best-performing combination with highest biodiesel yield.

**Combination**	**Biodiesel weight (g)**	**Biodiesel yield (%)**	**Mean yield (%)**
Casein + Glucose	0.079 ± 0.006	15.8 ± 1.2	15.8
* Casein + Glucose + Eggshell	0.135 ± 0.006	27.0 ± 1.2	27.0
$NaNO_3$ + Glucose	0.021 ± 0.006	4.2 ± 1.2	4.2
$NaNO_3$ + Glucose + Eggshell	0.123 ± 0.006	24.6 ± 1.2	24.6

Table S4. Gas chromatography mass spectrometry (GC–MS) profiling of fatty acid methyl ester composition under direct transesterification and eggshell-catalyzed transesterification of lipids from *Fusarium solani* 102F. Values represent relative peak area (%). RT, retention time.

RT (min)	Compound	Structure	Direct transesterification (Relative peak area %)	Eggshell-catalyzed transesterification (Relative peak area %)
6.33	Glycerol	$C_3H_8O_3$	—	2.61
21.59	Methyl tetradecanoate (C14:0)	$C_{15}H_{30}O_2$	—	0.68
25.67	Hexadecanoic acid, methyl ester (C16:0)	$C_{17}H_{34}O_2$	5.19	11.65
26.68	9-Octadecenoic acid (C18:1)	$C_{19}H_{36}O_2$	3.64	9.47
26.70	n-Hexadecanoic acid (C16:0)	$C_{16}H_{32}O_2$	18.08	—
27.03	Palmitic acid (C16:0)	$C_{16}H_{32}O_2$	—	1.04
28.68	9,12-Octadecadienoic acid, methyl ester (C18:2)	$C_{19}H_{34}O_2$	4.95	8.40
28.86	9-Octadecenoic acid, methyl ester (C18:1)	$C_{1n}H_{36}O_2$	20.12	32.72
29.42	Methyl stearate (C18:0)	$C_{1n}H_{38}O_2$	—	7.47
29.42	Tetradecanoic acid, 12-methyl-, methyl ester	$C_{16}H_{32}O_2$	1.70	—
29.80	cis-Vaccenic acid (C18:1N11)	$C_{19}H_{36}O_2$	42.23	19.60
29.80	Oleic acid (C18:1N9)	$C_{1n}H_{36}O_2$	—	19.62
30.34	9,12-Octadecadienoyl chloride	$C_{1n}H_{33}ClO$	4.07	3.38
41.24	6,9,12,15-Docosatetraenoic acid, methyl ester (C22:4)	$C_{23}H_{38}O_2$	—	0.33

Table S5. Two-way analysis of variance (ANOVA) testing the effects of carbon source, nitrogen source, and their interaction on lipid concentration (g/L) and lipid yield (%) produced by *Fusarium solani* 102F.

**Response**	**Source**	**F-statistic**	***P*-value**	η^2_p
Lipid concentration	Carbon	130,898	0.001	1.0
	Nitrogen	42,772	0.001	1.0
	Carbon × Nitrogen	22,24	0.001	1.0
Lipid yield	Carbon	56,963	0.001	1.0
	Nitrogen	25,437	0.001	1.0
	Carbon × Nitrogen	12,660	0.001	1.0

η^2_p, partial eta-squared.
